# Keyhole Limpet Hemocyanin-Based Multimodal Management in Two Dogs with Presumptive Urothelial Carcinoma: Long-Term Clinical and Imaging Follow-Up

**DOI:** 10.3390/vetsci13090878

**Published:** 2026-08-27

**Authors:** Ji-hee Hong, Jihyun Kim, Kun-Ho Song

**Affiliations:** 1 Department of Veterinary Internal Medicine, College of Veterinary Medicine, Chungnam National University, Daejeon 34134, Republic of Korea; jolivety@hanmail.net; 2 Top Care Animal Medical Center, Goyang 10500, Republic of Korea; ghobo213@naver.com

**Keywords:** dog, presumptive urothelial carcinoma, keyhole limpet hemocyanin, Immucothel, non-steroidal anti-inflammatory drug, selenium, case report

## Abstract

Canine urothelial carcinoma (UC) is commonly managed with non-steroidal anti-inflammatory drugs (NSAIDs), alone or combined with cytotoxic chemotherapy. Evidence supporting subcutaneous keyhole limpet hemocyanin (KLH) in canine UC is lacking. We describe two client-owned dogs with presumptive lower urinary tract UC managed with NSAIDs, KLH, and selenium after cytotoxic chemotherapy was declined. Case 1 was an 11-year-7-month-old castrated male Maltese with bladder-neck/proximal-urethral thickening. A positive veterinary bladder tumor antigen test, compatible serial imaging, and low-cellularity urine cytology showing suspected urothelial hyperplasia with dysplasia supported clinical suspicion; however, BRAF and BRAF-PLUS assays were negative and histopathology was unavailable. Case 2 was an 11-year-old neutered male Poodle with a BRAF V595E-positive urethrovesical-junction lesion on imaging, without histopathologic confirmation. Both dogs received NSAIDs, Immucothel^®^ (1 mg subcutaneously), intravenous sodium selenite (200 μg/kg), and oral selenium (100 μg/dog q24h). Serial CT and ultrasonography documented persistent localized abnormalities over prolonged follow-up, but measurements obtained by different modalities were not treated as directly comparable. Adverse events were assessed retrospectively and not prospectively graded using VCOG-CTCAE criteria. Because the diagnoses were presumptive and the interventions were concurrent and uncontrolled, the independent contribution, efficacy, and safety of KLH cannot be determined. These cases are descriptive and hypothesis-generating.

## Simple Summary

Urothelial carcinoma is the most common malignant tumor of the canine urinary bladder. This report describes two dogs with presumptive lower urinary tract urothelial carcinoma that underwent multimodal management with non-steroidal anti-inflammatory drugs, subcutaneous keyhole limpet hemocyanin (Immucothel^®^), and selenium supplementation after their owners declined cytotoxic chemotherapy. Long-term clinical, laboratory, and imaging observations are presented. Neither diagnosis was histopathologically confirmed, the dogs received several interventions, imaging assessment was not standardized, and keyhole limpet hemocyanin-specific immune responses were not measured. Therefore, the cases do not demonstrate efficacy or safety of any individual component. Their value is as detailed, hypothesis-generating clinical observations that may inform the design of prospective controlled studies.

## 1. Introduction

Urothelial carcinoma (UC), historically termed transitional cell carcinoma, is the most common malignant tumor of the canine urinary bladder and may also involve the urethra, prostate, or ureters [1,2,3,4]. Canine UC frequently arises in the trigone, bladder neck, or proximal urethra, where complete surgical excision is often not feasible. Clinical consequences include hematuria, pollakiuria, dysuria, urethral or ureteral obstruction, local invasion, and metastatic spread [1,2,3,4].

Medical management therefore plays a central role. NSAIDs with cyclooxygenase-inhibitory activity have documented antitumor activity in canine UC and are frequently used alone or in combination with agents such as mitoxantrone, vinblastine, carboplatin, or doxorubicin [3,5,6,7,8,9]. Although combination protocols can achieve meaningful tumor control, treatment selection in practice is influenced by age, concurrent disease, anticipated toxicities, cost, logistical burden, and owner preference.

Detection of the canine BRAF V595E mutation in urine or tissue provides strong molecular support for UC, but a negative result does not exclude the diagnosis because a proportion of canine UCs lack detectable BRAF V595E [10,11,12]. Urine cytology, bladder tumor antigen assays, ultrasonography, CT, and MRI may contribute to a clinical diagnosis, but none replaces histopathology in all cases. False-positive bladder tumor antigen results can occur with hematuria or inflammatory urinary tract disease, and low-cellularity cytology may be nondiagnostic.

Keyhole limpet hemocyanin (KLH) is a highly immunogenic molluscan protein capable of inducing humoral and cellular immune responses. KLH-based preparations have been evaluated in human non-muscle-invasive bladder cancer, usually as intravesical or adjuvant immunotherapy after tumor resection [13,14,15,16]. Those settings differ substantially from subcutaneous administration in dogs with naturally occurring lower urinary tract disease, and clinical evidence supporting this approach in canine UC is lacking.

This report describes the diagnostic basis, treatment delivery, and long-term clinical, laboratory, and imaging observations in two dogs with presumptive UC undergoing NSAID-, KLH-, and selenium-based multimodal management. The aim is descriptive and does not establish causality, comparative benefit, efficacy, or safety of any individual component.

## 2. Case Presentation

### 2.1. Treatment Rationale and Protocol

Both dogs were treated during routine clinical care. NSAID monotherapy and NSAID-based cytotoxic chemotherapy were discussed with the owners, including anticipated benefits, adverse effects, monitoring, and treatment burden. Cytotoxic chemotherapy was declined because of substantial owner concern regarding adverse effects. Tissue biopsy was not pursued because of the anatomically challenging lesion locations and the owners’ decision not to undertake an invasive procedure. The KLH/selenium regimen was then selected empirically as an individualized, off-label multimodal approach, with palliative goals of maintaining quality of life and monitoring clinically apparent progression. The owners were informed that this regimen had not been validated for canine UC.

Immucothel^®^ (immunocyanin, 1 mg; biosyn Arzneimittel GmbH, Fellbach, Germany) was administered subcutaneously in the right inguinal region adjacent to the inguinal lymph node. Injectable sodium selenite was administered off-label at 200 μg/kg IV using Selenase^®^ T pro injectione (1000 μg selenium/20 mL; biosyn Arzneimittel GmbH), diluted 1:1 with 0.9% saline and infused over approximately 40 min. Oral selenium was administered at 100 μg/dog PO q24h (one tablet containing 100 μg selenium; Cefak KG, Kempten, Germany). The regimen was extrapolated from limited anecdotal veterinary experience rather than a validated canine UC protocol; no peer-reviewed study establishes these doses as an anticancer regimen in dogs. No cytotoxic anticancer drug was administered.

### 2.2. Case 1

**Figure 1:**
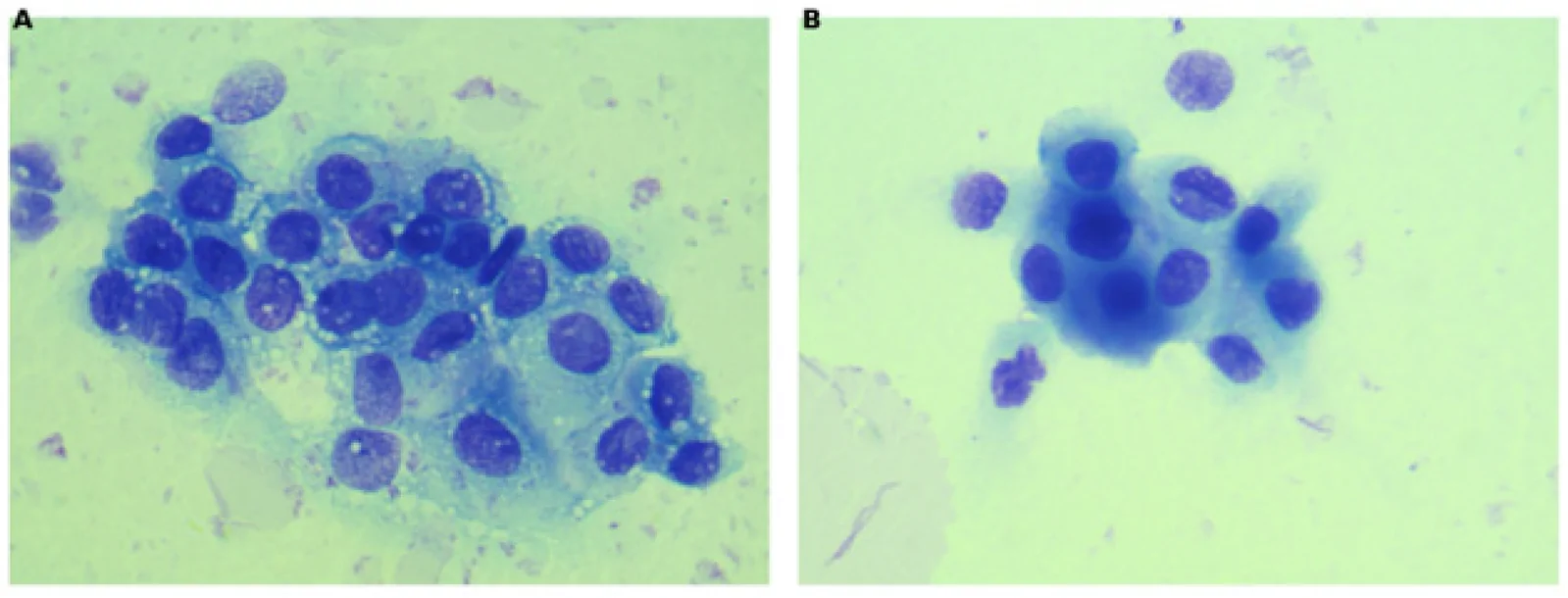
Representative urine cytology from Case 1. (**A**,**B**) Cohesive groups of urothelial cells showing anisocytosis, anisokaryosis, coarse chromatin, and occasional binucleation. The urine specimen was centrifuged, and a smear prepared from the resulting sediment was stained with Diff-Quik and examined by light microscopy. The specimen was of low cellularity and was interpreted as suspected urothelial hyperplasia with dysplasia; the findings were not diagnostic of malignancy. The original objective magnification could not be recovered from the retrospective medical record.

An 11-year-7-month-old castrated male Maltese weighing approximately 3.15 kg was first evaluated at Top Care Animal Medical Center on 22 February 2023. Approximately 4 months earlier, a lower urinary tract tumor had been suspected at another hospital, and a veterinary bladder tumor antigen (V-BTA) test was positive on 30 September 2022. The dog had received piroxicam for approximately 3 months and had been off the drug for approximately 3 weeks at presentation because satisfactory clinical improvement had not been observed. Mild discomfort during urination and dysuria were present; gross hematuria was not observed. At presentation, heart rate was 168 beats/min, rectal temperature was 39.7 °C, systolic blood pressure was 136 mmHg, mucous membranes were pink, and capillary refill time was <1 s. No clinically relevant cardiopulmonary abnormality or peripheral lymph-node enlargement was identified. CBC showed mild thrombocytosis (platelets, 537 × 10^3^/μL) without anemia or a clinically relevant leukocyte abnormality (hematocrit, 38.5%; white blood cells, 7.16 × 10^3^/μL). Serum biochemical findings included mildly increased blood urea nitrogen (32.4 mg/dL), mildly increased γ-glutamyl transferase activity (8.19 U/L), and mild hypophosphatemia (2.77 mg/dL), with creatinine (0.99 mg/dL), alanine aminotransferase activity (40 U/L), and SDMA (11 μg/dL) within their respective reference intervals. Urine was collected by urethral catheterization for BRAF testing and cytologic examination. Routine urinalysis, sediment examination, and urine culture were not performed; consequently, microscopic hematuria and concurrent urinary tract infection could not be retrospectively excluded. Both CADET^®^ BRAF and BRAF-PLUS assays were negative. Cytology, reported on 2 March 2023, showed a low-cellularity specimen containing urothelial cells with moderate anisocytosis, moderate-to-marked anisokaryosis, coarse chromatin, occasional binucleation, and up to two nucleoli; mitotic figures were not observed. The interpretation was suspected urothelial hyperplasia with dysplasia rather than definitive malignancy, and chronic inflammatory stimulation remained a differential diagnosis. Histopathology was not obtained because the bladder-neck/proximal-urethral location made biopsy technically challenging and the owner declined the procedure. Contrast-enhanced CT on 22 February 2023 showed an approximately 2-cm segment of mural thickening extending from the bladder neck into the proximal urethra, with maximal thickness of approximately 4.3 mm. No abdominal lymphadenopathy, adjacent pelvic invasion, or pulmonary metastasis was detected. Ultrasonography on the same date measured the lesion at approximately 6.75 mm. These CT and ultrasonographic values were not considered directly comparable because imaging plane, bladder distension, and lesion-margin definition differed (Figure 1).

Piroxicam was restarted on 22 February 2023 at 0.3 mg/kg PO q24h, with misoprostol for gastrointestinal protection. KLH and intravenous selenium were administered on 22 February, 8 March, 22 March, 5 April, 19 April, 3 May, 31 May, 1 July, and 29 July 2023. The first six treatments were separated by 14 days; the subsequent intervals were 28, 31, and 28 days. Oral selenium was given between visits as described above. The exact treatment and imaging chronology is summarized in Table 1.

**Figure 2:**
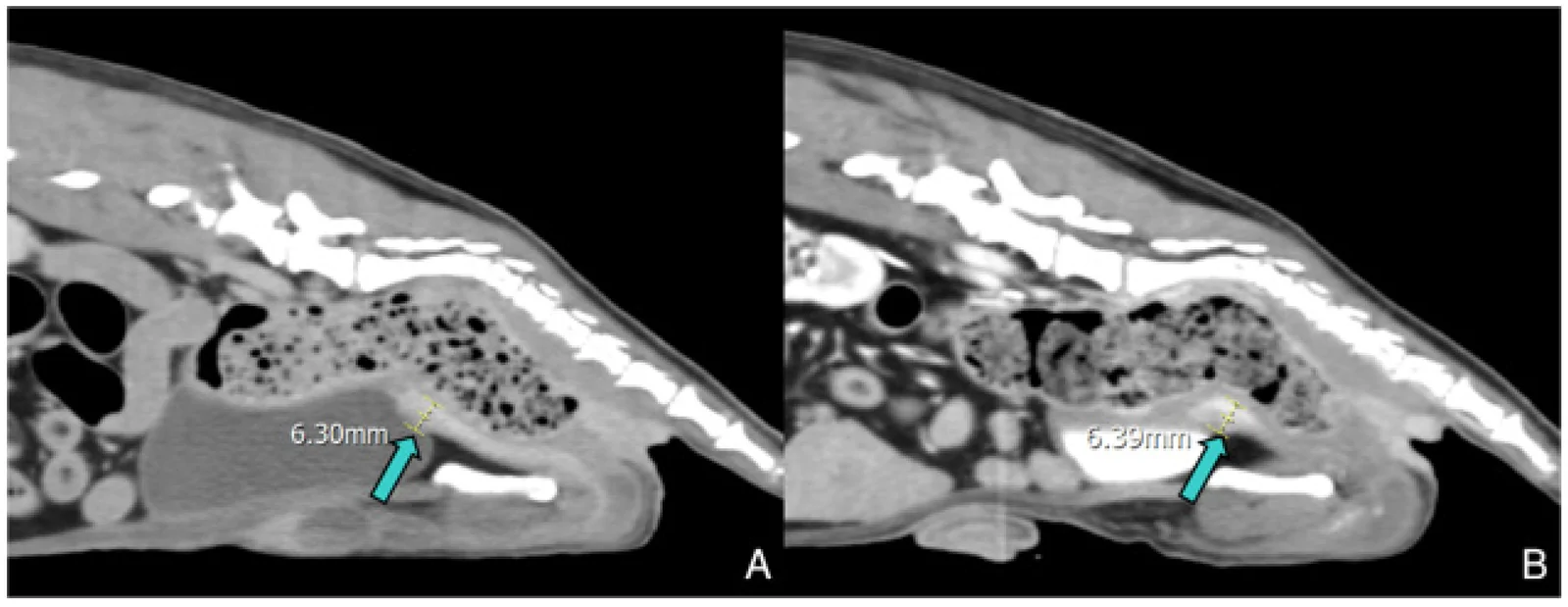
Representative sagittal CT images of the bladder-neck/proximal-urethral lesion in Case 1 on 23 August 2023. (**A**,**B**) Recorded lesion measurements were 6.30 and 6.39 mm, respectively. Colored arrows identify the measured lesion.

Serial imaging demonstrated persistent localized thickening. Follow-up CT on 23 August 2023 recorded maximal thickness of approximately 6.4 mm without thoracic metastasis or regional lymph-node enlargement. On 4 August 2024, CT measurements ranged from 4.05 to 4.09 mm, whereas same-day ultrasonography measured approximately 8.1 mm. Because these values were obtained using different modalities and planes and no standardized volumetric response criteria were applied, they were recorded descriptively and were not classified as regression, stable disease, or progression (Figure 2).

Mild left adrenal enlargement was also noted. An ACTH stimulation test on 29 December 2024 yielded a baseline cortisol concentration of 0.8 μg/dL and a post-ACTH concentration of 18.8 μg/dL. The result was considered equivocal, but no compatible clinical signs of hyperadrenocorticism were present; monitoring without adrenal-specific treatment was elected.

Serial CBC and serum biochemical examinations were reviewed retrospectively. Intermittent mild abnormalities included thrombocytosis early in follow-up, mild decreases in hematocrit, and isolated biochemical changes; no persistent abnormality was documented as requiring permanent discontinuation of the regimen. No immediate hypersensitivity reaction was documented in contemporaneous records during day hospitalization. Serum selenium concentrations were not measured, adverse events were not assessed using a predefined checklist, and VCOG-CTCAE grading was not performed prospectively; therefore, formal safety conclusions cannot be drawn.

Given the absence of histopathology, negative BRAF assays, low-cellularity non-definitive cytology, and the possibility of reactive urothelial change, the final classification was presumptive lower urinary tract UC rather than confirmed UC.

### 2.3. Case 2

An 11-year-old neutered male Poodle was evaluated after a lower urinary tract lesion had been detected during health screening. On 25 October 2023, ultrasonography at the referring hospital showed approximately 6.7 × 7.2 mm thickening near the bladder-urethral junction; no urinary signs were reported. On 6 August 2024, the dog remained without urinary signs, and repeat ultrasonography recorded a bladder nodule of 9.26 × 7.64 mm and bladder/urethral junction thickening of approximately 9.6 × 10.5 mm. A urine BRAF assay subsequently detected the canine BRAF V595E mutation. Cytology had reportedly been performed externally, but the original report and slides were unavailable for review. Histopathology was not obtained; the diagnosis was therefore presumptive BRAF V595E-positive UC.

At the initial treatment evaluation, CBC and serum biochemical results did not show anemia, thrombocytopenia, azotemia, or clinically relevant hepatopathy (hematocrit, 51.8%; white blood cells, 5.24 × 10^3^/μL; platelets, 250 × 10^3^/μL; blood urea nitrogen, 24.4 mg/dL; creatinine, 1.0 mg/dL; SDMA, 14 μg/dL; alanine aminotransferase activity, 42.2 U/L). Contrast-enhanced CT on 13 September 2024 identified an ill-defined enhancing urethrovesical-junction lesion of approximately 5 mm; ultrasonography measured 8.95 × 6.86 mm. No definitive regional or distant metastasis was identified at baseline.

By 13 September 2024, the referring hospital had changed the NSAID to firocoxib (5 mg/kg PO q24h). Firocoxib was being administered on 24 September and 8 October, was discontinued by 22 October and remained withheld on 5 November, was restarted on 3 December, and was continued through 15 April 2025. It was discontinued again by 27 May 2025 and remained discontinued through the final follow-up.

**Figure 3:**
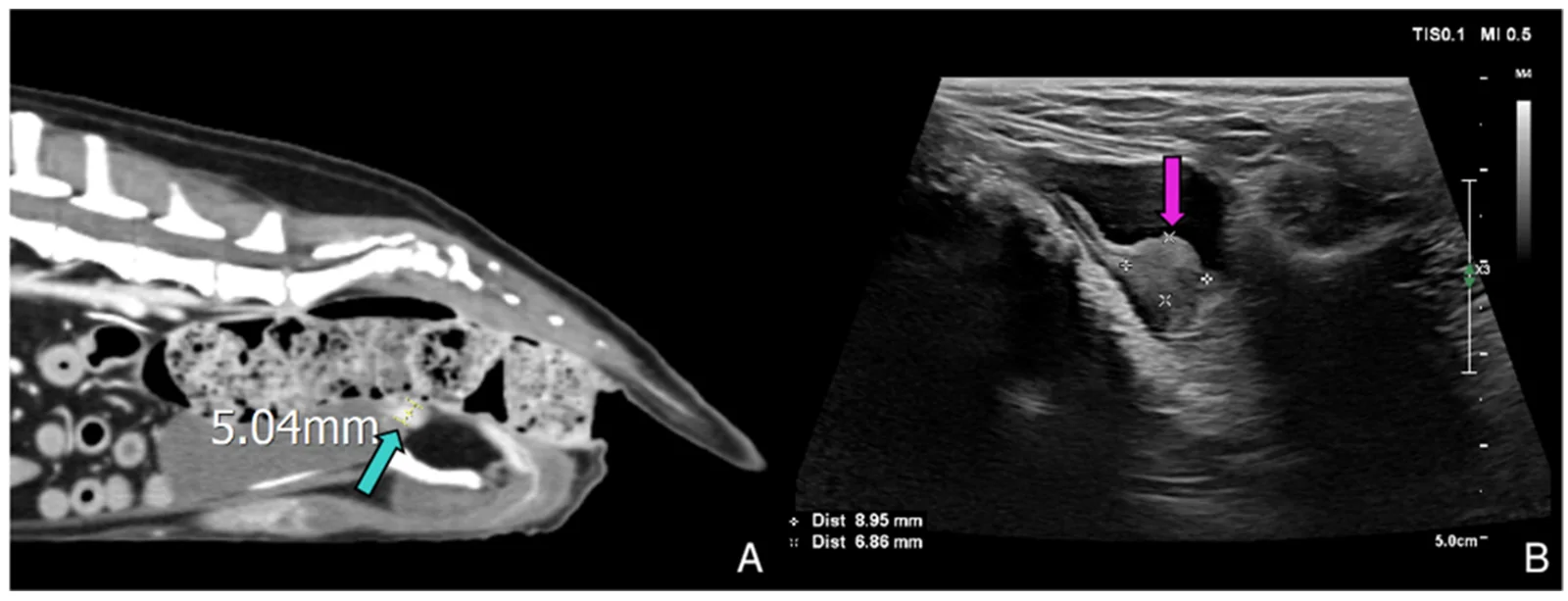
Baseline imaging of the urethrovesical-junction lesion in Case 2 on 13 September 2024. (**A**) CT measurement of approximately 5.04 mm. (**B**) Ultrasonographic measurement of 8.95 × 6.86 mm. Colored arrows identify the measured lesion. CT and ultrasonographic measurements were interpreted separately and were not considered directly comparable.

**Figure 4:**
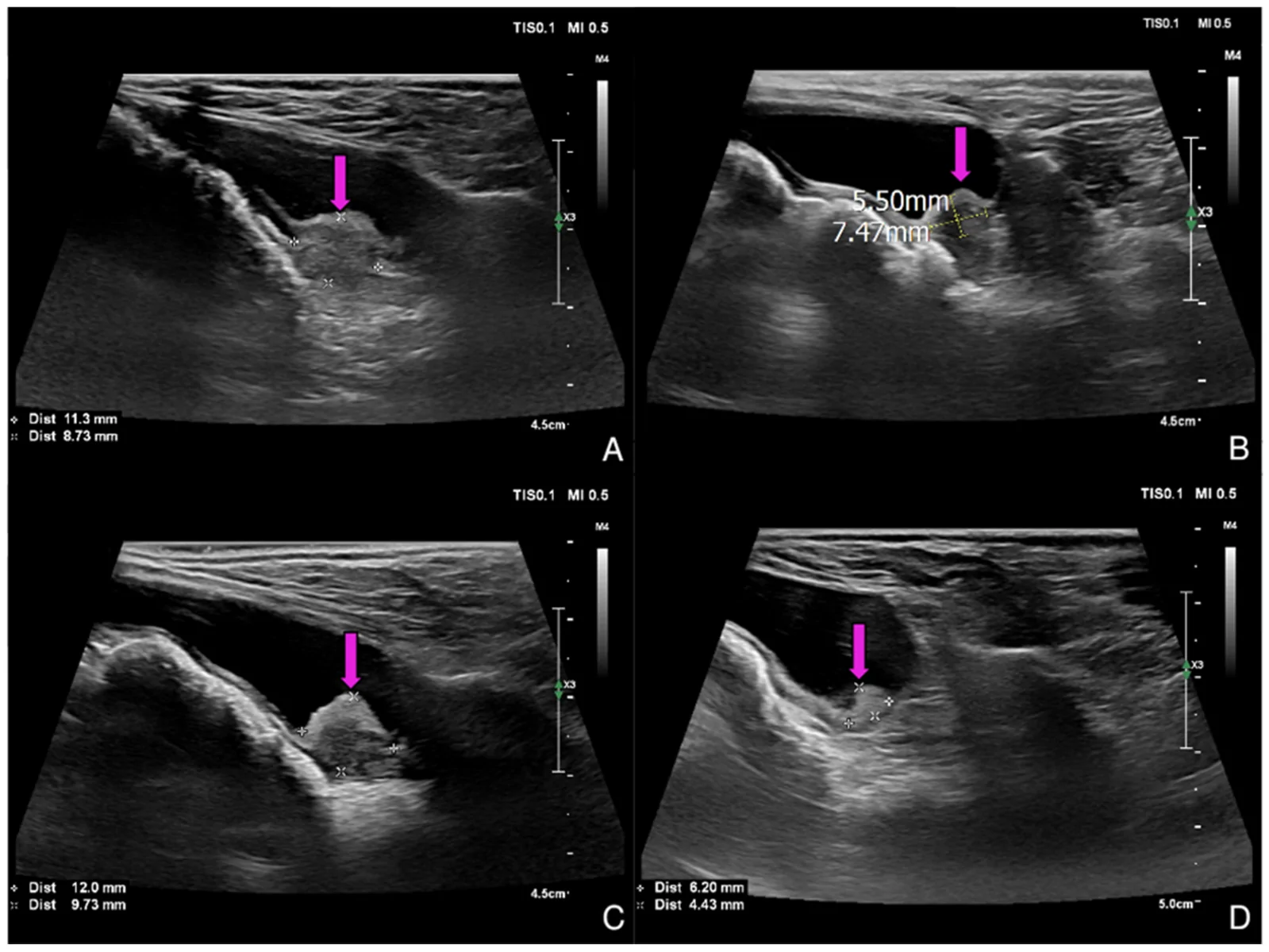
Serial ultrasonographic images of the urethrovesical-junction lesion in Case 2. (**A**) 3 December 2024, 11.3 × 8.73 mm. (**B**) 18 February 2025, 7.4 × 5.5 mm. (**C**) 15 April 2025, 12.0 × 9.7 mm. (**D**) 27 May 2025, 6.2 × 4.4 mm. Colored arrows identify the measured lesion. These measurements are descriptive and should not be interpreted as standardized tumor-response assessments.

KLH/intravenous selenium treatment dates were 24 September, 8 October, 22 October, 5 November, 3 December, and 27 December 2024; 21 January, 18 February, 15 April, 27 May, 8 July, 19 August, 30 September, 11 November, and 23 December 2025; and 3 February, 24 March, 12 May, 1 July, and 19 August 2026. A total of 20 cycles were documented. Actual intervals were 14 days for cycles 1–4, 24–28 days for cycles 5–8, 56 days between cycles 8 and 9, approximately 42 days for cycles 9–16, and 49–50 days for cycles 16–20 (Figure 3 and Figure 4).

The dates, imaging findings, NSAID status, and KLH intervals are summarized in Table 2. Where the exact NSAID date or a comparable imaging dimension was unavailable, this is stated rather than estimated.

On 13 May 2025, right-sided perineal herniation of the urinary bladder was identified, and corrective surgery was performed at the referring hospital. Subsequent ultrasonographic measurements were therefore interpreted cautiously because bladder position, imaging plane, and postoperative remodeling may have affected lesion measurement.

Serial CBC and serum biochemical examinations were reviewed during follow-up. Intermittent mild abnormalities occurred, but no clinicopathologic event was documented as requiring permanent discontinuation of the regimen. No immediate hypersensitivity reaction was documented in contemporaneous records; however, retrospective monitoring without prospective VCOG-CTCAE grading precludes a formal safety conclusion.

## 3. Discussion

These cases provide detailed long-term clinical, treatment, laboratory, and imaging documentation for two dogs with presumptive lower urinary tract UC undergoing KLH-based multimodal management. Their principal value is the description of clinical feasibility, exact treatment delivery, and prolonged follow-up in a setting where owners declined cytotoxic chemotherapy. They do not demonstrate that KLH, selenium, or the combined regimen produced tumor control or prolonged survival.

Diagnostic certainty differed between the dogs. Case 2 had BRAF V595E detection and compatible imaging, providing strong molecular support, but histopathology was unavailable. Case 1 had a positive V-BTA result, dysplastic urothelial proliferation on low-cellularity cytology, and persistent lower urinary tract thickening, but both BRAF assays were negative. A negative BRAF assay does not exclude UC [10,11,12]; nevertheless, reactive or inflammatory urothelial disease could not be definitively excluded. The qualifier “presumptive” is therefore used consistently for both cases.

NSAIDs are major confounders. Piroxicam and firocoxib have recognized antitumor activity in canine UC [5,6,7,8,9], and both dogs received NSAIDs during relevant portions of follow-up. Selenium was another uncontrolled intervention. Selenium participates in redox regulation and immune function, but has dose-dependent effects and a relatively narrow margin between nutritional adequacy and toxicity in dogs [17,18]. No validated intravenous or oral selenium anticancer dose exists for canine UC, and serum selenium, selenoprotein activity, and pharmacodynamic markers were not measured. The independent contributions of KLH, NSAIDs, and selenium therefore cannot be separated.

KLH is highly immunogenic, but no KLH-specific antibody titers, cytokine concentrations, lymphocyte responses, natural-killer-cell activity, or tumor immune markers were measured. Thus, these cases provide no direct evidence that a meaningful systemic or tumor-directed immune response occurred. Any proposed immunotherapeutic mechanism remains hypothetical.

Host epithelial responses to immune and inflammatory stimuli may include apoptosis, epithelial–mesenchymal transition (EMT), and tissue remodeling. Apoptosis can modify epithelial turnover, whereas EMT and related plasticity may influence invasion, immune interaction, and treatment resistance. Recent studies involving RBMX2 in Mycobacterium bovis-infected bovine lung epithelial cells illustrate links among infection-associated signaling, apoptosis, EMT, and cellular remodeling [19,20]. However, those models did not investigate canine UC or KLH. No tissue markers of apoptosis, EMT, or remodeling were evaluated here, and these concepts should not be interpreted as mechanisms demonstrated in either dog.

Serial CT and ultrasonographic measurements were not directly interchangeable. Bladder distension, imaging plane, operator technique, mural-versus-luminal margins, and lesion geometry may alter measured dimensions. In addition, perineal herniation and artifact limited CT evaluation in Case 2. Because standardized volumetric or RECIST-like response criteria were not applied, formal response categories were avoided. The absence of definitive metastasis at recorded examinations is clinically relevant but cannot be interpreted as prevention of metastasis.

Safety also cannot be established. Clinical observations and laboratory findings were obtained from retrospective medical records, monitoring schedules were not standardized, serum selenium was not measured, and adverse events were not graded prospectively using VCOG-CTCAE v2 [19]. Intermittent hematologic and biochemical abnormalities occurred, particularly in Case 2, and their relationship to KLH, selenium, NSAIDs, concurrent disease, or other treatment cannot be determined. The statement that no immediate hypersensitivity reaction was documented reflects only contemporaneous records and is not a prospective safety assessment.

Despite these limitations, the prolonged clinical courses, repeated subcutaneous administration, and longitudinal monitoring may help define questions for future research. A prospective study should require histopathology or predefined robust diagnostic criteria; standardize NSAID, KLH, and selenium exposure; use a suitable comparator; apply objective imaging and quality-of-life endpoints; prospectively grade adverse events; measure selenium status; and assess KLH-specific humoral and cellular immune responses.

## 4. Limitations

The major limitations are the retrospective uncontrolled design; inclusion of only two dogs; absence of histopathologic confirmation; weaker diagnostic certainty in Case 1; concurrent NSAID and selenium administration; an empirically derived off-label protocol; incomplete reconstruction of some NSAID and oral-supplement records; nonstandardized imaging and response assessment; lack of prospective toxicity grading; absence of immune and selenium-status monitoring; and risks of selection, survivorship, and publication bias. These limitations preclude conclusions regarding efficacy, comparative benefit, metastatic prevention, mechanism, or safety of KLH.

## 5. Conclusions

These two cases describe long-term clinical and imaging observations in dogs with presumptive urothelial carcinoma undergoing keyhole limpet hemocyanin-based multimodal management. Because the diagnoses were not histopathologically confirmed and NSAIDs and selenium were administered concurrently, the independent contribution, efficacy, and safety of keyhole limpet hemocyanin cannot be determined. These observations are hypothesis-generating and support further prospective controlled investigation rather than the use of keyhole limpet hemocyanin as an alternative to established treatment.

## Figures and Tables

**Table 1:** Case 1 chronological treatment and imaging summary.

Date	Modality/Event	Recorded Lesion Finding	NSAID Status	KLH Cycle/Interval
30 September 2022	V-BTA	Positive	Piroxicam subsequently administered at referring hospital	Before KLH
22 February 2023	CT	4.3 mm maximal thickness; ~2-cm affected segment	Piroxicam restarted 0.3 mg/kg q24h	Cycle 1; baseline
22 February 2023	US	6.75 mm	Piroxicam	Cycle 1; baseline
8 March 2023	Clinical visit	No standardized tumor measurement recorded	Piroxicam	Cycle 2; 14 days
22 March 2023	US	6.4 mm	Piroxicam	Cycle 3; 14 days
5 April 2023	Clinical visit	No standardized tumor measurement recorded	Piroxicam	Cycle 4; 14 days
19 April 2023	US	6.9 mm	Piroxicam	Cycle 5; 14 days
3 May 2023	US	7.2 mm	Piroxicam	Cycle 6; 14 days
31 May 2023	US	Approximately 6.9 mm	Piroxicam	Cycle 7; 28 days
1 July 2023	US	Approximately 6.9 mm	Piroxicam	Cycle 8; 31 days
29 July 2023	US	Approximately 6.9 mm	Piroxicam	Cycle 9; 28 days
23 August 2023	CT	Approximately 6.4 mm; no definitive metastasis	Piroxicam	25 days after cycle 9
14 January 2024	US	Approximately 7.5 mm	Piroxicam	No further KLH documented
4 August 2024	CT	4.05–4.09 mm	Piroxicam	No further KLH documented
4 August 2024	US	Approximately 8.1 mm	Piroxicam	Same-day CT/US; not directly comparable
December 2024	Clinical decision	Piroxicam discontinued	Discontinued	No further KLH documented
23 June 2026	US	Approximately 6–7 mm; no documented obstruction	Off NSAID	Long-term follow-up

**Table 2:** Case 2 chronological treatment and imaging summary.

Date	Modality/Event	Recorded Lesion Finding	NSAID Status	KLH Cycle/Interval
25 October 2023	US	6.7 × 7.2 mm	No NSAID documented	Before KLH
6 August 2024	US	9.26 × 7.64 mm bladder nodule; ~9.6 × 10.5 mm junction lesion	No NSAID documented	Before KLH
13 September 2024	CT/US	CT ~5.0 mm; US 8.95 × 6.86 mm	Firocoxib	Baseline
24 September 2024	US	9.1 × 6.6 mm	Firocoxib	Cycle 1; 11 days
8 October 2024	US	10.7 × 7.0 mm	Firocoxib	Cycle 2; 14 days
22 October 2024	US	10.7 × 7.0 mm	Firocoxib discontinued	Cycle 3; 14 days
5 November 2024	US	10.7 × 7.0 mm	Firocoxib withheld	Cycle 4; 14 days
3 December 2024	US	11.3 × 8.73 mm	Firocoxib restarted	Cycle 5; 28 days
27 December 2024	US	10.2 × 8.5 mm	Firocoxib	Cycle 6; 24 days
21 January 2025	US	9.1 × 6.3 mm	Firocoxib	Cycle 7; 25 days
18 February 2025	US	7.4 × 5.5 mm	Firocoxib	Cycle 8; 28 days
18 March 2025	US	9.4 × 6.7 mm	Firocoxib	No KLH
15 April 2025	US	12.0 × 9.7 mm	Firocoxib	Cycle 9; 56 days
13 May 2025	Clinical/imaging event	Bladder herniation; surgery at referring hospital	Not specified	No KLH
27 May 2025	US	6.2 × 4.4 mm	Firocoxib discontinued	Cycle 10; 42 days
8 July 2025	US	7.6 × 4.9 mm	Firocoxib discontinued	Cycle 11; 42 days
19 August 2025	US	4.9 × 3.0 mm	Firocoxib discontinued	Cycle 12; 42 days
30 September 2025	US	4.9 × 3.0 mm	Firocoxib discontinued	Cycle 13; 42 days
11 November 2025	US	4.0 × 3.0 mm	Firocoxib discontinued	Cycle 14; 42 days
23 December 2025	US	4.0 × 3.0 mm	Firocoxib discontinued	Cycle 15; 42 days
3 February 2026	US	4.0 × 3.0 mm	Firocoxib discontinued	Cycle 16; 42 days
24 March 2026	US	4.0 × 3.0 mm	Firocoxib discontinued	Cycle 17; 49 days
12 May 2026	US	4.0 × 3.0 mm	Firocoxib discontinued	Cycle 18; 49 days
1 July 2026	US	4.0 × 3.0 mm	Firocoxib discontinued	Cycle 19; 50 days
19 August 2026	US	4.0 × 3.0 mm	Firocoxib discontinued	Cycle 20; 49 days

## Data Availability

The original contributions presented in this study are included in the article. Further inquiries can be directed to the corresponding author.
